# The Proximity Prediction Hypothesis: How predictive coding of CT-touch explains Autonomous Sensory Meridian Response and its therapeutic applications

**DOI:** 10.3389/fnbeh.2025.1688172

**Published:** 2025-10-24

**Authors:** Josephine R. Flockton, Catherine E. J. Preston, Cade McCall

**Affiliations:** ^1^Department of Psychology, University of York, York, United Kingdom; ^2^Touch and Pain Lab, Department of Psychology, University of York, York, United Kingdom; ^3^Lifelike Lab, Department of Psychology, University of York, York, United Kingdom

**Keywords:** Autonomous Sensory Meridian Response (ASMR), predictive coding, interoception, peripersonal space, C-tactile afferents, transcutaneous auricular vagus nerve stimulation (taVNS), anxiety, autism

## Abstract

Autonomous Sensory Meridian Response (ASMR) is a pleasant tingling sensation felt across the scalp and neck, widely reported to reduce anxiety and improve sleep. The Proximity Prediction Hypothesis (PPH) is the first comprehensive predictive coding model explaining ASMR’s underlying neural mechanism. PPH posits that near-field acoustic cues from common ASMR triggers (e.g., brushing sounds, whispered speech) engage the audio-tactile Peripersonal Space Network, generating a top-down prediction of gentle C-tactile (CT) touch on CT fibre-rich skin of the scalp and neck. This prediction suppresses locus coeruleus (LC) arousal and increases vagal output, offering a mechanistic explanation for the phenomenon’s therapeutic benefits. In a subjective-experience survey (*N* = 64), ASMR-labelled trials were rated significantly more pleasant but only slightly more arousing than controls. Pleasantness predicted both the presence and intensity of tingles, supporting PPH’s core claim that hedonic value, rather than sympathetic activation, drives the graded somatosensory response. PPH situates ASMR within the Neurovisceral Integration framework, predicting measurable Central Nervous System-Autonomic Nervous System (CNS-ANS) markers (beta-band desynchronisation in the posterior insula and proportional increases in high-frequency heart rate variability with tingle intensity). It further predicts reduced LC activity during ASMR, stronger effects in individuals with high interoceptive prediction error (e.g., anxiety, autism), and attenuation of tingles when spatial proximity cues are removed. By integrating auditory proximity, CT-touch anticipation, and autonomic regulation into a single predictive-coding account, PPH provides a unified, testable framework for explaining ASMR, offering a blueprint for translating this sensory phenomenon into targeted, evidence-based interventions for anxiety and sleep disorders.

## 1 Introduction

Autonomous Sensory Meridian Response (ASMR) is a sensory phenomenon characterised by a pleasant tingling sensation felt across the scalp and often moving down the back of the neck, elicited by very specific stimuli. The sensation is triggered by auditory and/or audiovisual cues. Sounds that induce ASMR are varied and broad ranging, but the most popular triggers are slow, whispered speech, rhythmic hair brushing and tapping sounds (Barratt and Davis, 2015; Fredborg et al., 2021; Poerio et al., 2018). Alongside this, ASMR is also elicited via videos on media sharing platforms like YouTube where content creators use objects or their own voices to produce sounds that trigger the response in listeners. This is done by placing the camera and microphone near to the performers’ mouths or hands while they whisper or manipulate objects to make noises into the microphone. Over the past decade, ASMR content has transitioned from a niche phenomenon to a mainstream YouTube staple. As of 2022, there were approximately 500,000 ASMR-focused channels and an estimated 25 million ASMR videos on the platform, illustrating the breadth and scale of its cultural reach. Many ASMR videos fall into role-play genres that simulate close personal attention, including hairdresser visits, spa treatments, makeup application, doctor’s appointments, and other interpersonal care scenarios.

This popularity is seemingly driven by perceived benefits from experiencing the ASMR phenomenon, which go beyond the initial pleasant sensation. Survey work with hundreds of viewers found that 98% reported using ASMR for relaxation, 82% to help fall asleep, and about 70% to reduce stress or anxiety (Barratt and Davis, 2015, *N* = 475). In laboratory follow-ups, participants who experience tingles report lower state-anxiety scores and improved mood up to thirty minutes after listening (Fredborg et al., 2021) suggesting the phenomenon provides more than just a pleasant distraction during the tingling experience itself and offers longer term affective benefits to those who enjoy it. These self-reports have also been scaffolded by physiological evidence. In a within subjects study that compared tingling to non-tingling segments of the same videos, Poerio et al. (2018) found a reliable heart rate deceleration accompanied by an increase in high frequency heart rate variability (HF-HRV). HF-HRV is a widely accepted non-invasive index of parasympathetic nervous system activity, often associated with states of calm and relaxation. Specifically, greater HF-HRV reflects increased vagal influence on the heart, indicating a shift toward physiological rest and recovery.

Recent work by Hozaki et al. (2025) extends this evidence using finger photoplethysmography (PPG), which not only captures pulse rate but also pulse wave amplitude, a measure of peripheral blood flow and vascular tone. In their study, both ASMR and nature videos reduced pulse rate relative to baseline, but ASMR produced significantly greater reductions. Moreover, ASMR was associated with increased pulse wave amplitude, consistent with peripheral vasodilation. Because vasodilation reflects parasympathetic dominance over vascular tone, these PPG findings complement HR and HRV evidence by demonstrating that ASMR’s autonomic effects extend beyond cardiac regulation to include vascular relaxation, supporting the interpretation of ASMR as inducing a coordinated parasympathetic shift. These parasympathetic-shift indicators are consistent with reduced sympathetic outflow, but the interpretation that ASMR down-regulates tonic locus coeruleus (LC) activity remains inferential. PPG cannot directly index LC firing, and the observed combination of bradycardia and vasodilation is best understood as a physiological profile compatible with reduced LC tone, rather than definitive evidence. Future work could test this pathway more directly. For example, pupillometry offers a non-invasive proxy for LC activity, with pupil diameter shown to covary with LC firing in humans (Murphy et al., 2014). Neuromelanin-sensitive MRI and LC-targeted fMRI approaches can provide in-vivo markers of LC integrity and activity (Betts et al., 2019; Trujillo et al., 2023), allowing individual differences in ASMR-related parasympathetic shifts to be linked with LC dynamics. Pharmacological modulation also provides a causal testbed: reducing LC output (e.g., with α2-agonists such as clonidine) should potentiate ASMR-related vagal indices, whereas elevating noradrenergic tone would be expected to blunt them (Wang et al., 2014). Together, such approaches would allow a more rigorous evaluation of whether the LC-vagus axis mediates the parasympathetic profile observed during ASMR.

Although most research on ASMR has focused on mood benefits, some survey studies have revealed that sleep improvement is also a strong motivation for listening in many people. In Barratt and Davis’s (2015) 475 participant survey, 82% of responders reported using ASMR videos “often” or “always” to fall asleep faster. A later large-scale online study (Smejka and Wiggs, 2022; *N* = 1,037) found that ASMR viewing improved relaxation and mood across participants who did and did not suffer from insomnia. Although improvements were strongest in those who experienced tingles, no significant differences emerged between insomniacs and other groups in their response magnitude.

A mechanistic account is needed to link three disparate elements of the ASMR phenomenon: the acoustic character of the triggers, the subjective percept of pleasant scalp tingles, and the body-wide calming represented by physiological correlates like HRV and PPG, as well as reported mood and sleep benefits. A natural starting point is the Neurovisceral Integration (NVI) Model (Thayer and Lane, 2000). NVI frames mental state regulation as an interaction between the cortical central-autonomic network (CAN) and subcortical autonomic nuclei. When this interaction is smooth, indexed by high vagal tone and HF-HRV, the organism is flexible and resilient; when it is disrupted, anxiety and rumination flourish. Within this hierarchy the locus coeruleus functions as a noradrenergic “gain knob”; meaning elevated tonic LC firing biases the body toward sympathetic readiness, whereas a drop in LC tone likely lifts inhibition over the dorsal-motor nucleus of the vagus (DMV) and permits parasympathetic dominance, and calm. In a way, the LC and the vagus operate a seesaw-like regulatory axis that modulates perception and bodily state between arousal and relaxation. Here, the term “arousal” is used in two related but distinct senses: (i) tonic vigilance, determined largely by baseline LC activity, and (ii) stimulus-specific activation, such as pupil dilation or SCR, reflecting transient orienting to an input. The PPH framework speculates that both occur in sequence during ASMR; a brief orienting arousal phase, followed by parasympathetic accommodation when the cue is integrated as affiliative. Existing ASMR findings, such as HRV increase during tingling and subjective experience reports, fit this framework, implying vagal activation and a downshift in LC tone. Yet no published stepwise neural model currently explains how auditory stimuli like whispers or brushing sounds could initiate this regulatory shift, let alone generate a tingling sensation across the scalp as a consequence.

Despite the range of auditory triggers that can elicit ASMR in listeners, one property which they arguably all have in common is that they can be categorised as proximal, near-ear stimuli, rich in spatial cues, illustrated by three key acoustic features shown across the literature. First, very large interaural level differences (ILDs) and sub-millisecond interaural time differences (ITDs) signal that the sound source is only a few centimetres from the listener’s head. ASMR YouTube video recordings are typically made with binaural “dummy-head” microphones whose fake pinnae and ear canals preserve these cues; playback over loudspeakers reduces them, but headphones, through which 90% of listeners choose to experience ASMR (Barratt and Davis, 2015; *N* = 475), deliver them unchanged, recreating the illusion that a hand or brush is at the ear. Second, the spectrum is colour-shifted by head-shadowing, meaning high frequencies above 8 kHz roll off steeply in the contralateral ear, a cue which listeners tend to interpret as indicating close spatial proximity (Begault and Trejo, 2000). Third, ASMR content creators often favour slow amplitude envelopes and low overall sound pressure levels. This means that the loudness of the signal rises and falls gradually, over hundreds of milliseconds or more, rather than in sharp, percussive bursts. A whispered phrase, a brush stroke across a microphone, or a series of soft taps typically shows a smooth, rounded waveform without abrupt transients. In addition, keeping the overall sound pressure level low ensures the audio remains intimate and non-startling, helping listeners maintain a relaxed, parasympathetic state; louder levels would recruit the middle ear reflexes and risk activating the sympathetic “alerting” system, which would contradict the calming goal of ASMR.

These findings converge on an interesting idea, that ASMR stimuli may work to convince the auditory system that an object is virtually approaching or touching the ear or scalp, in the absence of any real physical contact. A mechanistic model must therefore account for the special spatial signature of these sounds, then explain how such proximity information could cascade into both the tingling percept and the parasympathetic shift measured in HRV and through reported improvements in mood and sleep. This paper proposes a Proximity Prediction Hypothesis (PPH) to integrate the audio-tactile features mentioned above, with the NVI framework, arguing that near-field sounds pre-activate the brain’s Peripersonal Space Network and prompt a top-down prediction of impending gentle CT-touch on the scalp.

Valtakari et al. (2019) observed that ASMR experiences are accompanied by pupil dilation, while Poerio et al. (2018) reported increased skin conductance responses (SCR) during tingling segments compared to control periods. Both pupil dilation and SCR are well-established markers of sympathetic nervous system activity, indicating that ASMR is not a purely parasympathetic phenomenon. This has caused some debate in the literature, given its reportedly calming profile. However, as McGeoch and Rouw (2020) note, the combination of heart rate deceleration and increased SCR suggests both sympathetic and parasympathetic involvement and, because eccrine sweat glands (underlying SCR) receive only sympathetic innervation, while the heart is dually innervated by both sympathetic and parasympathetic pathways, the net decrease in heart rate points to an overall shift toward increased vagal tone. This aligns with the PPH model, in which pupil dynamics in ASMR are predicted to reflect a transition from orienting to affiliative calm, where near-ear cues initially engage the LC-noradrenaline system, producing a transient pupil dilation to enhance sensory gain. As peripersonal space and CT-afferent touch predictions converge, tonic LC activity is suppressed and parasympathetic output dominates in the model, leading to heart rate deceleration, increased HF-HRV, feelings of calm, and we predict, eventual pupil constriction—a hypothesis that is yet to be tested in future research. This biphasic pattern would accommodate both sympathetic (early attentional) and parasympathetic (later calming) components, supporting the interpretation of ASMR as a flow state (Peifer et al., 2014) of “relaxed alertness” characteristic of safe, affiliative proximity.

This biphasic profile can also be interpreted as reflecting an initial mismatch between perception and reality; where the brain briefly treats the near-ear cue as if physical contact were imminent, engaging orienting and sympathetic resources. A subsequent “accommodation” phase might follow, in which the system recognises the safety and affiliative value of the stimulus, allowing parasympathetic dominance to emerge. In this way, early sympathetic activation is not contradictory to ASMR’s calming effects but may be a necessary precursor, sharpening sensory gain before the vagal system restores balance.

After explaining the theoretical background, current evidence in the area will be collated and assessed in the context of the PPH model. Then, we report original illustrative survey data from sixty-four listeners in an immersive ASMR listening study, demonstrating that hedonic valence drives the tingling experience and its intensity, thus providing empirical support for the PPH model. Clinical applications and the reported benefits to mental health and sleep in ASMR experiencers will be discussed with the PPH model and CNS-ANS integration in mind. Future research will be suggested to test the theory, with falsifiable predictions for findings across CNS-ANS research, encompassing heart rate variability, pupil-indexed LC dynamics, and beta band neural signatures, in behavioural, EEG, and MEG studies, if the model is to be supported.

## 2 Theoretical foundations

### 2.1 The interoceptive brain and predictive coding

According to Interoceptive Predictive Coding accounts (Critchley and Harrison, 2013; Barrett and Simmons, 2015), cortical areas generate continuous, probabilistic forecasts (or “priors”) about what the viscera, skin, and muscles should feel like. Incoming afferent data are compared with these priors and any difference found is the prediction error signal (Feldman and Friston, 2010). A close match is desirable; a mismatch registers as physiological surprise and, when sustained chronically, has been linked to heightened anxiety (Paulus and Stein, 2010). When the incoming signal and priors match (or the error is negligible), this implies that the sensory world is unfolding as expected. Most of this comparison takes place in areas such as the posterior and anterior insula, which influence autonomic nuclei in the brainstem. The posterior insula receives raw interoceptive input, constructs a sensory map of the body, and forwards that map to the anterior insula, where predictions and errors are integrated with the affective context (Critchley and Harrison, 2013). When the match between the prior and signal is close, and the prediction error is small to negligible, for example, if you feel the gentle pressure that you expected while holding a cup in your hand, the anterior insula sends an inhibitory signal to the locus coeruleus (LC), the brainstem hub for noradrenaline release. In simple terms, this inhibits the LC’s usual role in promoting arousal and vigilance. As tonic LC firing drops, its noradrenergic brake on the dorsal-motor nucleus of the vagus (DMV) is lifted. The result is increased vagal output and a rise in high frequency heart rate variability (HF-HRV), the parasympathetic signature of calm suited for rest, digestion, and affective ease (Samuels and Szabadi, 2008).

This low precision gate explains everyday illusions like in the phantom phone buzzing phenomenon, where people report feeling their phone vibrate even when it is not; a strong learned prior (“my phone is about to vibrate”) meets either minimal somatic noise or no detectable cutaneous input at all. Because any residual error is labelled as low precision, the posterior insula fills in the expected buzz with a somatosensory echo, a phantom vibration, the anterior insula reports “prediction fulfilled,” and the LC-vagus axis remains calm (Lin et al., 2013). Virtual reality touch has a similar mechanism where viewing a virtual stick stroking a forearm that you associate with your own body in virtual reality produces tingles in 89% of users despite zero skin input on their actual arm in real life, because the visual prior overwhelms the ill-defined cutaneous error (Pilacinski et al., 2023), it is more likely that you are being touched and it is light and not hugely noticeable, than that all other, more reliable, priors are wrong in anticipating that touch when your previous experience and the visual input suggests it is very likely. In both cases of touch illusions, the visual or contextual prior overwhelms the ambiguous tactile input. The cue is interpreted as consistent with expected gentle touch but not clear enough to generate high precision error, allowing the prior to dominate. Touch is considered ill-defined in these circumstances because the sensory evidence is either absent, ambiguous, or delivered through a channel (e.g., auditory or visual) that does not strongly engage tactile precision mechanisms. When this occurs, the brain is more likely to accept the predicted sensation and resolve the ambiguity in favour of the expected state.

Crucially, “precision”, the brain’s estimate of sensory reliability, i.e., its confidence in the fidelity of a particular sensory channel, modulates how much any given error matters. High precision channels (e.g., retinal contrast, a pin-prick sensation) deliver errors that are hard to ignore; low precision channels however (faint rustling, diffuse light pressure) deliver errors that can be treated as background noise. Here we suggest that, when the brain issues a strong top-down prior like “I am about to feel a gentle stroke” and the incoming signal is fuzzy, delayed, or absent, the mismatch is labelled as low precision. In that case the posterior insula may simply fill in the expected sensation itself and send a “prediction fulfilled” message upstream. Because the error never gains salience, the anterior insula does not escalate to the LC, tonic LC firing falls, and the vagal brake is released even though no physical touch ever occurred.

**Figure 1:**
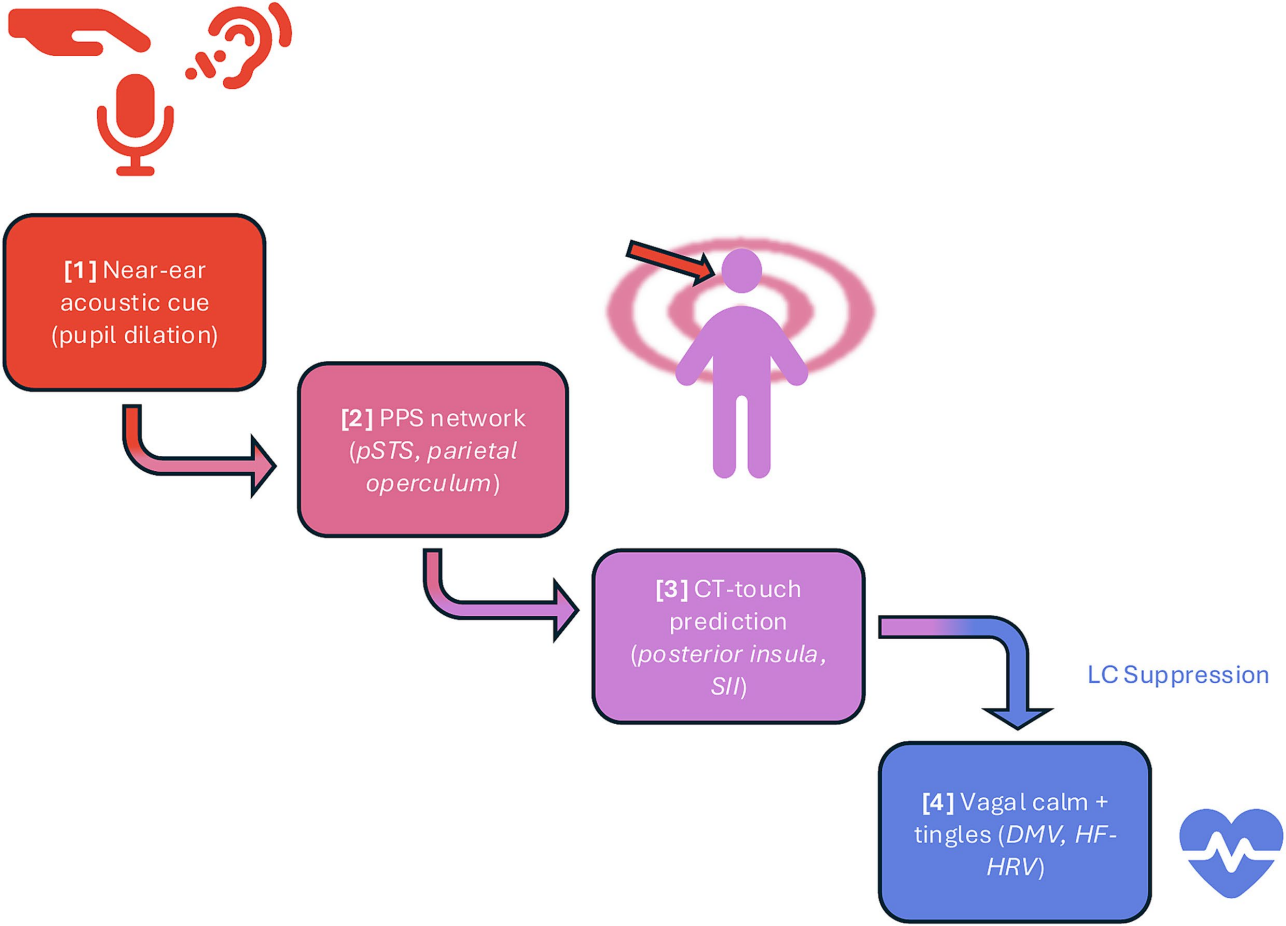
Proposed cascade of the Proximity Prediction Hypothesis (PPH). A near-ear sound activates peripersonal space (PPS) networks, which forwards a C-tactile (CT) touch prediction to somatosensory and interoceptive regions; confirmation of that prediction suppresses locus coeruleus (LC) tone, disinhibits the vagal system, and generates parasympathetic calm and tingling sensations. [1] Near-ear acoustic cue → PPS detection: binaural whispers, tapping, and brushing sounds carry strong interaural time and level differences, interpreted by the posterior superior temporal sulcus (pSTS) and adjacent areas as proximal, human-origin sounds (Schürmann et al., 2006; Belin et al., 2000; Warren and Griffiths, 2003); at this early orienting stage, sympathetic attentional mechanisms such as pupil dilation are transiently recruited to enhance sensory gain (Valtakari et al., 2019). [2] PPS network → CT-touch prediction: pSTS and parietal operculum project to the posterior insula and secondary somatosensory cortex (SII), simulating tactile consequences of perceived social proximity, especially on CT-rich scalp/neck regions (Löken et al., 2009; Gazzola and Keysers, 2009). [3] Accurate prediction → LC suppression and vagal disinhibition: minimised prediction error reduces anterior insula drive to the LC, lowering tonic noradrenaline and lifting inhibitory control over the dorsal motor nucleus of the vagus (DMV), increasing parasympathetic tone and yielding cardiac deceleration and increased high-frequency HRV (Paulus and Stein, 2006; Samuels and Szabadi, 2008). [4] Conscious correlate; the tingles: pre-activation of insula/SII yields a synchronous, spatially diffuse cortical volley experienced as a tingling somatosensory echo of predicted contact. Icons (ear, hand, microphone, person, circular shapes, heart rate icon) from Font Awesome Free, licenced under CC BY 4.0; edited for size, colour, and orientation.

It is important to note, however, that not all mismatches will be labelled low precision from the outset. When an ambiguous sensory cue first enters the system, for instance, a near-ear sound suggesting touch without any corresponding cutaneous input, the brain may briefly treat this as a salient error. In predictive coding terms, this transient up-weighting of error signals recruits the LC-noradrenaline system, manifesting as a short-lived sympathetic orienting phase (indexed by pupil dilation or SCR)—evidence for this comes from several converging studies. Although much of the direct LC physiology comes from primate electrophysiology, these findings have been foundational for broader cross-species models of arousal. Aston-Jones and Cohen (2005) showed that phasic LC activity functions as an orienting response to novel or behaviourally significant events, while Dayan and Yu (2006) framed phasic norepinephrine release as a neural interrupt signal marking unexpected uncertainty, i.e., prediction errors with high precision. Similarly, Sara and Bouret (2012) demonstrated that LC activity underlies rapid shifts in arousal when attention is reoriented to unexpected stimuli. Together, these accounts support the idea that the first stage of the proposed ASMR cascade may involve a sympathetic “alerting” phase driven by prediction error, before the system reclassifies the error as low precision and accommodates it. Once this occurs, the present theory suggests that the anterior insula inhibits tonic LC firing and parasympathetic dominance emerges, explaining the biphasic pattern of initial orienting followed by vagal calm. This series of predictive, neurophysiological events, from sensory prior to vagal activation, forms the basis of that theory, the Proximity Prediction Hypothesis (PPH) cascade, a stepwise model proposed to explain how the characteristic calm and tingling response of ASMR can arise from purely auditory cues. Each element of this cascade is explored in subsequent theoretical sections and visualised in Figure 1.

A similar process is proposed more generally in the Somatic Error Hypothesis (Khalsa and Feinstein, 2019), where the brain reduces prediction error by generating bodily sensations that match an expected state. While this mechanism is typically invoked to explain chronic symptoms in somatising disorders, here we extend its logic to a benign interoceptive illusion felt by those who experience ASMR.

### 2.2 The audio-tactile fabric of peripersonal space

Prediction in this case does not operate in isolation, it is shaped by multisensory maps of Peripersonal Space (PPS), which can be thought of as a region of 20–30 cm space surrounding the body where approaching objects are most likely to make contact. Importantly, PPS is not a simple distance gradient; it behaves like a biological boundary. Stimuli presented just inside the bubble elicit abrupt neural and behavioural changes, whereas equally small decrements in distance once the stimulus is outside the peripersonal space have little effect (Làdavas and Serino, 2008; Serino et al., 2015). A substantial body of multisensory work shows that the brain treats a near-ear sound as a potential touch event.

Early single-unit electrophysiology in macaque monkeys revealed a class of multisensory neurons in ventral premotor and parietal regions, including the ventral intraparietal area (VIP), that integrate tactile, visual, and auditory signals relevant to peripersonal space. Auditory cues alone can activate neurons in peripersonal space-sensitive regions, including the VIP, for instance, Graziano et al. (1999) reported that broadband noise sources moving toward the head, from 70 cm to 10 cm, caused multisensory neurons in VIP to fire more vigorously than when those same stimuli moved within far space. This indicates that approaching sounds, even in the absence of visual input, can signal potential contact and recruit defensive spatial coding. Moreover, Avillac et al. (2007) demonstrated that VIP neurons integrate visual and tactile input when sensory events are spatially and temporally aligned. These neurons often integrate tactile and auditory information, reinforcing the idea that auditory proximity cues are biologically relevant indicators of incoming contact.

Importantly, VIP neurons respond to stimuli that occur both on the body (i.e., within a neuron’s tactile receptive field) and just beyond it, typically within a few tens of centimetres. This alignment of visual and somatosensory receptive fields reflects a body-centred coding of nearby space, a neural basis for anticipating contact (Colby et al., 1993; Rizzolatti et al., 1997).

Magnetoencephalography supports the idea that ASMR-like stimuli can activate such somatosensory regions. Schürmann et al. (2006) played realistic sounds resembling haircut and water-dripping scenarios and found beta-band desynchronisation in the secondary somatosensory cortex (S2). This effect is supported across broader studies. Canzoneri et al. (2012) found that sounds approaching the hand significantly accelerated tactile responses once perceived within peripersonal space. A meta-analysis by Holmes et al. (2020) confirmed a modest (15 ms) reduction in tactile reaction times when sounds occurred near the body versus farther away, although they noted variability and small effect sizes. Additionally, Taffou and Viaud-Delmon (2014) demonstrated that looming “rough” sounds, those with threat-like acoustic properties, expanded the effective PPS boundary, triggering tactile facilitation at greater distances than smoother sounds. Together, these findings support the notion that sound proximity is a potent modulator of sensory integration and may help explain how ASMR content elicits embodied responses despite being purely auditory.

These findings demonstrate that the posterior STS, inferior parietal cortex, and the parietal operculum behave like proximity detectors, amplifying their response when an auditory object crosses the PPS boundary, and is therefore likely to make physical contact. This supports the notion that sound proximity is a potent modulator of sensory integration and may explain how ASMR content elicits embodied responses despite being purely auditory. Within this framework, PPS responses could be generating a transient orienting mismatch, when a stimulus is detected inside the boundary without accompanying tactile confirmation. This mismatch could recruit sympathetic arousal to heighten vigilance, but once sensory prediction resolves in favour of a safe, affiliative source, parasympathetic accommodation then follows. ASMR may therefore harness this sequential PPS dynamic, beginning with an alerting phase and culminating in vagal release. Such proximity-sensitive firing is proposed to represent the first node in the PPH cascade, the moment when the brain interprets near-ear sounds as predictive of imminent affective touch, triggering downstream autonomic changes detailed in the next sections (see Figure 1).

### 2.3 C-tactile afferents and the mechanism behind affective touch

If, as PPH suggests, ASMR is occurring through prediction of affective touch, it is important to consider what exactly the brain is predicting and how that links to the reported ASMR experience. When contact does occur on the skin, it is detected by at least two tactile channels. Fast, myelinated A-*β* fibres handle discriminative features, conveying facts about the touch, like location, texture, and force, whereas C-tactile (CT) afferents are slow, unmyelinated fibres that overwhelmingly tend to innervate hairy skin regions. Microneurography shows that CT afferents respond optimally to gentle stroking at 1–10 cm s^−1^, with a firing peak at around 3 cm s^−1^, which is exactly the velocity of social grooming strokes in primates (Löken et al., 2009; Ackerley et al., 2014). Their firing rate predicts subjective pleasantness and drives oxytocin release, posterior-insula activation and a parasympathetic drop in heart rate (Ackerley et al., 2014; Pawling et al., 2017).

Human CT afferents have been recorded in scalp, face, forearm, abdomen and thigh areas (McGlone et al., 2014) and show a clear preference for hairy skin. While detailed follicle density maps are scarce, regions such as the scalp midline, nape, and upper back are widely associated with social grooming in primates and are plausible candidates for dense CT innervation (McGlone et al., 2014). These zones are therefore likely to be particularly well populated by CT-touch fibres. They are also prime cortical targets for affective touch, where the brain predicts a gentle, grooming-like sensation to land. Crucially, this is indeed where the ASMR tingling sensation is reported to be localised: the scalp, face, neck, and upper back (Barratt and Davis, 2015; Poerio et al., 2018; Lochte et al., 2018).

Behavioural data echo the physiology; in barbary macaques, bouts of allogrooming (a prosocial behaviour where animals of the same species groom one another) lower basal cortisol and heart rate within minutes (Shutt et al., 2007). In humans, five minutes of scalp massage at CT-optimal velocity produces a significant HF-HRV increase and self-reported anxiety reduction in Spielberger state-anxiety test scores (Diego and Field, 2009). Consistent with this, a 45 min relaxation massage before bed has been shown to enhance sleep efficiency in individuals with insomnia (Ntoumas et al., 2025). Moreover, meta-analytic evidence indicates that interventions involving head touch specifically, such as face or scalp massage, may confer particularly strong physical and mental health benefits (Packheiser et al., 2024), reinforcing the potential relevance of affective touch to ASMR-related somatosensory modulation. As the second stage of the cascade, CT-touch predictions anchor the brain’s expectation of safety and interpersonal care. Taken together, these findings establish CT-touch as a hedonic, anxiolytic, and sleep-promoting modality, and identify the scalp and neck as privileged substrates for such contact, exactly the locations where ASMR listeners report feeling their tingles.

One complementary, structural account of ASMR has been offered by McGeoch and Rouw (2020), who propose that ASMR may involve synesthetic cross-activation between the primary auditory cortex (A1) and affective-touch maps in the dorsal posterior insula (dpIns). Earlier functional evidence by Lochte et al. (2018) supports this coupling: in ASMR experiencers, moments of tingling elicited elevated BOLD activation not only in auditory and somatosensory regions but also in the nucleus accumbens and mPFC, implicating reward and affiliative circuitry in the perceptual experience. This suggests that ASMR may recruit not just tactile-sensory prediction routes but also reward/bonding networks. Under proximal, interpersonal conditions, near-ear sounds may recruit such regional cross-activation to simulate gentle social touch, triggering posterior-insula activity, activating reward/affiliative circuits, and promoting vagal engagement. Unlike the PPH, however, these accounts do not address the state-dependent gating, peripersonal space integration, or temporal autonomic cascade that determine *when* and *how* this cross-activation occurs. The two perspectives can therefore be viewed as complementary, with cross-activation describing the same plausible neural route (i.e., A1 to dpIns, and then on to reward/affiliative circuits) and the PPH specifying the predictive coding logic and dynamic conditions under which that route is engaged in the ASMR phenomenon. Furthermore, while McGeoch and Rouw’s hypothesis and Lochte’s findings link auditory input to affective touch and reward areas, they do not specify the computational mechanism by which tingles emerge, nor how such activation alone would produce the distinct, wave-like somatosensory echo characteristic of ASMR. The PPH extends this by proposing the predictive coding process and time-resolved neural signature capable of transforming such cross-activation into the tingling percept itself. To our knowledge, the PPH draws upon and extends these key models but represents the first explicit attempt in the literature to explain the ASMR tingling percept via a predictive coding account, linking sensory priors, insular prediction errors, and downstream autonomic responses.

### 2.4 The social neurocognitive context of ASMR

If ASMR indeed reflects a prediction of affiliative touch, then understanding the social and cognitive conditions that shape those priors becomes crucial. ASMR triggers overwhelmingly reflect socially salient acts like whispering, soft-spoken instruction, and gentle, attentive behaviours, many of which imply close interpersonal proximity. These cues may be sufficient to evoke predictions of touch-like feedback, particularly in individuals predisposed to interpret such signals as affiliative or comforting.

Recent empirical studies have identified five principal ASMR trigger categories, all of which share a perceptual association with human interaction: (1) viewing individuals interact with objects, (2) watching socially intimate acts, (3) hearing soft repetitive sounds, (4) simulated social interaction, and (5) whispering or chewing (Smith et al., 2020; Fredborg et al., 2018). Even seemingly nonsocial triggers, such as tapping or crinkling, often co-occur with goal-directed behaviours that implicitly suggest a human source (McErlean and Banissy, 2017). This convergence supports the idea that ASMR is scaffolded by social perceptual priors, often concerning caregiving or affiliative intent.

Recent work by Poerio et al. (2023) developed the ASMR Trigger Checklist (ATC), a validated tool for systematically identifying and categorising common ASMR triggers, to assess how individuals respond to a wide range of sounds. They found considerable variability in which triggers reliably induced tingles across participants. Critically, the most potent triggers tended to be those that implied gentle, interpersonal interaction or close physical proximity, such as whispering or soft tapping. This variability is consistent with the precision-weighting mechanism proposed by the PPH; individuals may assign higher predictive value to particular sensory cues based on their internal priors about social intent, interpersonal closeness, or expected affective touch. The ATC therefore offers a structured way to assess which auditory signals carry predictive weight in different individuals, and why the same cue may trigger ASMR in one person but not another. These findings reinforce the notion that ASMR emerges from a socially grounded predictive model shaped by prior experience, attachment tendencies, and interoceptive sensitivity.

Most significantly for the PPH, Poerio et al. (2023) study also showed that physical touch itself, rather than sound or visual cues, was not only the most commonly endorsed ASMR trigger reported to elicit a tingling sensation in participants (98%) but also the most intense, with minimal variation across individuals; the ATC subset of tactile and interpersonal triggers gave examples like “close-up movements directed at you” and “light touch on your face, e.g., make-up application”. This highlights that touch itself, whether anticipated or actively experienced is a core trigger for the ASMR tingling sensation, making the idea of a somatosensory echo even more plausible as it is clear that the tingling sensation is a ground truth for the phenomenon, not an abstract, novel response the brain is predicting. This emphasises that the tingles are less of a bodily illusion, as some may argue, and more of a plausible sensory prediction based on what it does actually feel like when people are really being touched.

Importantly, Poerio and colleagues argue that online ASMR content should be seen as a simulation of real-world interpersonal encounters rather than as distinct from them, and that trait ASMR may be meaningfully defined by a person’s sensitivity to touch-related triggers. In this way, their work empirically supports the idea that ASMR operates through predictive interoceptive mechanisms shaped by tactile expectation and affiliative social context. Consistent with this view, Gillmeister et al. (2022) demonstrated that gentle social touch enhanced the intensity and pleasantness of ASMR responses, but only in ASMR-experiencers, reinforcing the role of trait-dependent priors for affiliative interaction in driving the ASMR response. This final phase of the cascade, the culmination of proximity, touch prediction, and arousal regulation, is therefore likely shaped by an individual’s social priors, attachment style, and interoceptive sensitivity.

Neuroimaging studies further reinforce the social grounding of ASMR. Lee et al. (2020) found that during ASMR experiences, participants showed activation in brain regions implicated in social cognition and mental state simulation, including the posterior cingulate cortex, superior and middle temporal gyri, and the lingual gyrus. These areas are key components of the brain’s social mentalizing network, suggesting that ASMR may engage the same systems we use to interpret and internalise others’ intentions; particularly when those intentions are perceived as caring, attentive, or intimate.

Earlier work by Lochte et al. (2018) proposed that ASMR may function as a vestigial grooming response, with Lochte going on to suggest that ASMR may be a polymorphic trait, a term used in evolutionary biology to describe a characteristic that is present in some individuals of a species but not all, due to genetic or developmental variability. Common examples include wisdom teeth or lactose tolerance, traits that were once adaptive, but are now only expressed in certain subsets of the population. If ASMR is indeed a polymorphic vestige of an ancestral grooming response, this could explain why only some individuals report experiencing tingles in response to specific stimuli. Again, aligning with the PPH model’s suggestion that ASMR emerges only when an individual’s internal predictive model assigns high precision to interpersonal proximity cues, a tendency that may itself vary across individuals based on neurocognitive, social, or interoceptive traits. These accounts offer an ethological framework for why ASMR stimuli elicit pleasure and calm in a specific subset of individuals.

That subset may be defined, in part, by individual differences in trait empathy and sensory-emotional inhibition. McErlean and Banissy (2017) reported that ASMR experiencers tend to score higher on “Empathetic Concern”, suggesting a heightened sensitivity to social-affective cues. Others have found that ASMR is associated with reduced functional connectivity in the prefrontal cortex and default mode network (Smith et al., 2017; Fredborg et al., 2021), implying diminished top-down inhibition of incoming sensory-affective stimuli. In predictive coding terms, such individuals may assign greater precision to exteroceptive social cues while allowing these predictions to unfold with minimal suppression, creating fertile ground for ASMR to emerge.

### 2.5 Individual differences in ASMR

A consistent theme across the ASMR literature is the striking individual variability in both susceptibility and trigger potency. Not everyone experiences tingles, and those who do, often have different personal preferences for effective triggers. Using the ASMR Trigger Checklist (ATC), Poerio et al. (2022) showed that responses to different triggers are relatively stable within individuals but highly idiosyncratic across the population; whispering and soft tapping were amongst the most reliable elicitors, while other sounds such as chewing or eating were inconsistent and could even be aversive. This heterogeneity is echoed in misophonia, an intolerance for specific sounds (often human oral/nasal sounds like chewing or breathing) that reliably evoke strong negative emotional reactions (e.g., anger, disgust) and autonomic arousal in many people (Edelstein et al., 2013). Notably, some misophonia triggers overlap with ASMR triggers (e.g., chewing and other mouth sounds), therefore, the same cue can be reported as intensely aversive by some listeners yet induce pleasant tingling in others. McGeoch and Rouw (2020) argued that ASMR and misophonia can be seen as opposing outcomes of auditory-affective processing, with one yielding affiliative calm, the other defensive aversion depending on the preferences of the listener.

The PPH naturally accommodates such variability within a predictive coding framework. In ASMR experiencers, near-ear cues are weighted as affiliative priors, reducing insular prediction errors and downregulating LC-noradrenaline tone. In others, the same cues may be assigned negative priors, heightening error and sympathetic arousal, as in misophonia. This provides a mechanistic explanation for why identical auditory inputs can generate diametrically opposed affective outcomes.

Individual differences in social–emotional traits further moderate this process. Those who report experiencing strong ASMR tend to score higher on empathic concern (McErlean and Banissy, 2017) and exhibit reduced prefrontal and default-mode network connectivity (Smith et al., 2017; Fredborg et al., 2021), suggesting that greater sensory-affective permeability may support ASMR proneness. Conversely, individuals with atypical interoception or altered affective empathy, such as those with autism or anxiety, may experience either enhanced benefits or blunted responses, depending on how their predictive models weigh affiliative cues—a topic that will be explored further in section 6.2 of this paper. Attachment style may also play a role: early caregiving experiences calibrate priors about the reliability and comfort of close interpersonal contact (Mikulincer and Shaver, 2010). Securely attached individuals may be more likely to interpret ASMR cues as soothing and affiliative, whereas those with avoidant or anxious attachment might assign lower precision or even aversive value to the same signals.

Taken together, ASMR should be understood not as a uniform response but as a polymorphic trait (Lochte et al., 2018), expressed in some individuals but not others, shaped by differences in priors, attachment style, interoceptive processing, and sensory-emotional inhibition. Recognising this variability is essential both for theory, by preventing overgeneralisation, and for clinical translation, where personalisation will be necessary to ensure that interventions based on ASMR do not inadvertently provoke discomfort or aversion.

## 3 The role of pleasantness in ASMR

If a near-ear whisper is effective because it forecasts a slow, pleasant interpersonal contact, then the strength of the ASMR response should depend on how rewarding that predicted contact feels, not on its sheer acoustic energy. Within the PPH framework, pleasantness (valence) is expected to determine two outcomes: whether a listener classifies a segment as ASMR at all, and how intense the tingles feel during the ASMR experience. This mirrors genuine affective touch, where C-tactile firing rates track subjective pleasantness (Löken et al., 2009) and hedonic ratings predict downstream effects on pain perception (Pawling et al., 2017). By analogy, ASMR tingles should scale with pleasantness because the posterior insula propagates stronger predictions of affective touch when the hedonic prior is stronger.

This valence-first logic is also illustrated by the content ecology of ASMR. The most watched videos on YouTube are spa, hairdresser, and make-up roleplays in which creators whisper reassurances, move brushes and scissors centimetres from the microphone, and enact a caretaking script. Such clips are maximising both near-field spatial cues and a social-grooming context, forming a strong hedonic prediction with minimal arousal load. Because the CT-touch prediction is intrinsically hedonic, a dominance of pleasantness over arousal would mirror the physiology of real affective touch. Microneurography shows that the firing rate of C-tactile afferents rises monotonically as stroking speed approaches the 3 cm s^−1^ optimum and that subjective pleasantness ratings track this firing curve with an almost unit slope (Löken et al., 2009). Follow-up psychophysics demonstrated a similar scaling for behavioural impact; in Pawling et al. (2017) each one-point increase on a 10-point pleasantness scale produced an additional 0.9-point decrease in pain rating during concurrent heat stimulation, confirming that the more pleasant the predicted stroke, the stronger its sensory-affective consequence. In other words, CT-touch intensity is modulated by valence in exactly the way PPH predicts ASMR might be.

This account is further supported by recent behavioural data from Gillmeister et al. (2022), who found that ASMR responders reported significantly greater tingle intensity and pleasantness ratings in response to auditory ASMR triggers when accompanied by gentle interpersonal touch, whereas non-responders showed no such modulation. Notably, the strength of the tingle correlated more with pleasantness than with arousal, underscoring the centrality of hedonic predictions in driving ASMR’s intensity. Their findings align with the PPH in suggesting that social touch cues amplify ASMR not through generic arousal, but through affective reward mechanisms that may be trait-dependent.

At this point, it is useful to clarify how “arousal” can be defined; in some contexts, arousal refers to a general state of vigilance or sympathetic readiness (baseline tonic LC activity), while in others it denotes stimulus-specific activation, i.e., the subjective energetic quality evoked by a cue (Aston-Jones and Cohen, 2005; Sara and Bouret, 2012; Dayan and Yu, 2006). The PPH highlights that ASMR appears to involve both: a brief orienting arousal response [as seen during pupil dilation by Valtakari et al. (2019)] during the initial prediction error phase, followed by a lower-intensity, stimulus-specific activation that co-occurs with pleasant tingling and parasympathetic calm (reflected in self-reports and both heart rate deceleration and HF-HRV increase; Poerio et al., 2018).

Importantly, this does not mean that arousal is irrelevant to ASMR. Some triggers may increase both pleasantness and arousal, and the role of arousal remains equivocal. What PPH predicts, however, is that pleasantness will be the primary driver of whether a sound crosses the tingle threshold and of how strong those tingles become.

The next section tests this prediction directly, using trial-level behavioural data on pleasantness, arousal, and ASMR reports from an original survey dataset by the authors.

## 4 Illustrative behavioural evidence

### 4.1 Method

#### 4.1.1 Participants

Undergraduate students (*N* = 64) from the University of York took part. Recruitment did not require prior experience of ASMR, to avoid expectation bias while still allowing inclusion of those who had previously engaged with ASMR content. All participants were over 18 in age, gave informed consent, and none reported adverse reactions to ASMR sounds. It should be noted that this sample was restricted to undergraduate students, which may limit the generalisability of findings to other age groups or clinical populations.

#### 4.1.2 Stimuli

The auditory stimuli were drawn from a larger experiment in which these same participants had taken part. The present section focuses solely on the behavioural survey data.

The stimuli comprised of 18 sound clips: 13 experimental sounds intended to plausibly elicit ASMR (e.g., paper folding, tapping, stroking, brushing) created by a professional ASMR content creator (Nader Jaime, 2023), plus 5 control sounds (ambient traffic noise) presented via Sennheiser HD280 Pro Dynamic Hi-Fi Stereo headphones, as 5 s sound clips embedded within the online survey. Participants completed the survey within a sound-attenuated room to minimise distraction. Mouth sounds were deliberately excluded to avoid inadvertently triggering misophonia, though this reduces ecological validity given that chewing and whispering are both major triggers for many ASMR viewers.

#### 4.1.3 Procedure

Participants listened to 5 s clips of the13 ASMRtist-created experimental sounds and the 5 control sounds mentioned above, during a digital questionnaire assessing their subjective responses to the experimental stimuli. For each sound, participants were asked whether they believed they experienced ASMR (“Yes” or “No”). The participants were informed of the definition of ASMR in the information sheet provided, and there was no requirement to have been familiar with ASMR or know if you could experience it, to sign up for the study. If “Yes” was selected to suggest ASMR had been experienced for any sound, participants were prompted to provide a retrospective estimate of tingle intensity on a 0–10 scale, if they could recall the sensation. All participants also used on-screen sliders to rate the pleasantness and arousal associated with each sound on continuous scales from −250 (extremely unpleasant or calming) to +250 (extremely pleasant or arousing) whether they experienced ASMR for that sound or not. The survey was completed immediately after the a separate EEG experiment where the participants had listened to longer versions of all sound clips, while participants remained in the sound-proof testing room environment, to minimise memory decay and distraction, and using the same headphones for sound clip delivery. Not all participants provided tingle intensity ratings for each sound, as this question was optional and conditional on an ASMR report as well as their memory of it.

This retrospective design was chosen to avoid interrupting the listening session itself and has precedent in accepted foundational ASMR studies, where both survey (Barratt and Davis, 2015; Smejka and Wiggs, 2022) and laboratory work (Poerio et al., 2018) have relied on post-exposure reports to capture ASMR experiences. While immediate post-exposure ratings mitigate memory bias, they remain vulnerable to under- or over-estimation compared with real-time capture so this limitation should still be considered when interpreting the results.

#### 4.1.4 Statistical analysis

To investigate what drives whether a sound elicits ASMR, and the strength of the associated tingling sensation, mixed-effects regression models were implemented in R (version 4.4.0) using the lme4 (Bates et al., 2015), lmerTest (Kuznetsova et al., 2017), and broom.mixed (Bolker et al., 2022) packages. Predictors were z-scored to aid interpretation and comparability. A logistic mixed-effects model was used to predict ASMR classification (either Yes or No) from pleasantness and arousal ratings, with random intercepts for participant and sound. A subsequent model tested whether the effect of pleasantness on reported ASMR experience was moderated by arousal using an interaction term.

For trials where participants reported experiencing ASMR and rated its intensity, a linear mixed-effects model was used to predict tingle strength from pleasantness and arousal, again including an interaction term in a follow-up model. Visualisations were created using ggplot2 (Wickham, 2016) and ggeffects (Lüdecke, 2018), with predicted probability heatmaps and scatter plots depicting the effects of predictors across trials and sound clips.

### 4.2 Results

#### 4.2.1 What drives the ASMR decision?

A mixed-effects logistic regression model was fit with ASMR classification (Yes or No) as the outcome and z-scored pleasantness and arousal as fixed effects, with random intercepts for participant and sound.

Pleasantness emerged as a strong positive predictor of ASMR reports (*β* = 2.07 ± 0.23, *z* = 8.86, *p* < 0.001), corresponding to an odds ratio (OR) = 7.90 with a 95% CI = [5.00, 12.45], i.e., each 1 SD increase in pleasantness increased the odds of reporting ASMR by ~8×. While arousal showed a non-significant negative trend (*β* = −0.29 ± 0.17, *p* = 0.092; OR = 0.75, 95% CI = [0.53, 1.05]). This suggests that hedonic valence, rather than activation level, primarily drives the ASMR decision.

An interaction term between pleasantness and arousal was also tested to assess whether arousal modulated the effect of pleasantness. However, the interaction was not statistically significant (*β* = 0.20 ± 0.15, *p* = 0.194; OR = 1.22, 95% CI = [0.91, 1.64]), and did not improve model fit (likelihood ratio test: χ^2^(1) = 1.68, *p* = 0.195). Therefore, the probability of classifying a sound as ASMR was strongly driven by pleasantness across the full arousal range. Model performance indices were: AIC = 552.71, BIC = 579.55, *R*^2^ (marginal) = 0.279, *R*^2^ (conditional) = 0.767, ICC = 0.677, indicating substantial between-participant/sound clustering with a sizeable fixed effects contribution.

**Figure 2:**
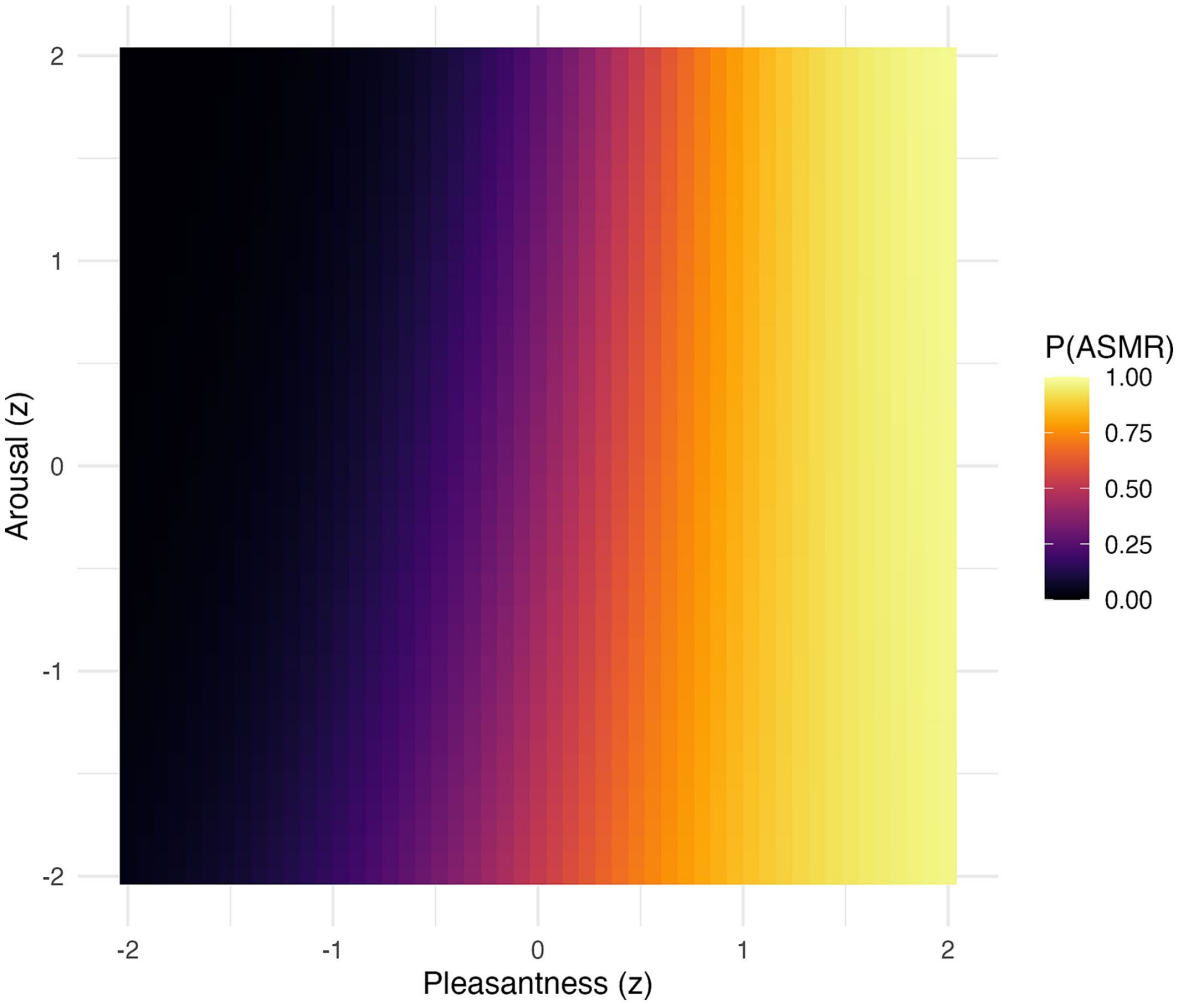
Predicted probability of ASMR classification as a function of z-scored pleasantness (x-axis) and arousal (y-axis). Colours show predicted probabilities from a logistic mixed-effects model with random intercepts for participant and sound (*N* = 64). Near-vertical contour lines indicate pleasantness as the dominant predictor, with minimal modulation by arousal.

Figure 2 visualises the predicted probability of reporting ASMR as a function of z-scored pleasantness and arousal. The near-vertical gradient in predicted probabilities underscores the dominance of hedonic valence in the ASMR decision; increases in pleasantness robustly predict ASMR reports across the full arousal range, while arousal adds minimal predictive power.

Table 1 presents the fixed effect estimates from the full logistic regression model, including the interaction term.

**Table 1:** Fixed-effect estimates from the logistic mixed-effects model predicting ASMR classification from z-scored pleasantness and arousal (including their interaction), with random intercepts for participant and sound.

Term	Estimate	SE	*Z*	*p*	95% Cl (*β*)	OR	95% Cl (OR)
(Intercept)	−0.499	0.424	−1.18	0.239	[−1.330, 0.332]	0.607	[0.264, 1.39]
Pleasantness (p_z)	2.070	0.233	8.86	0.000	[1.610, 2.520]	7.90	[5.00, 12.45]
Arousal (a_z)	−0.293	0.173	−1.69	0.091	[−0.633, 0.047]	0.746	[0.531, 1.048]
Pleasantness x Arousal (p_a:a_z)	0.196	0.151	1.30	0.194	[−0.100, 0.492]	1.22	[0.905, 1.636]

Odds ratios (OR) with 95% CIs are shown. Model fit: AIC = 552.71, BIC = 579.55; *R*^2^ (marginal) = 0.279, *R*^2^ (conditional) = 0.767; ICC = 0.677.

#### 4.2.2 Tingle intensity

**Figure 3:**
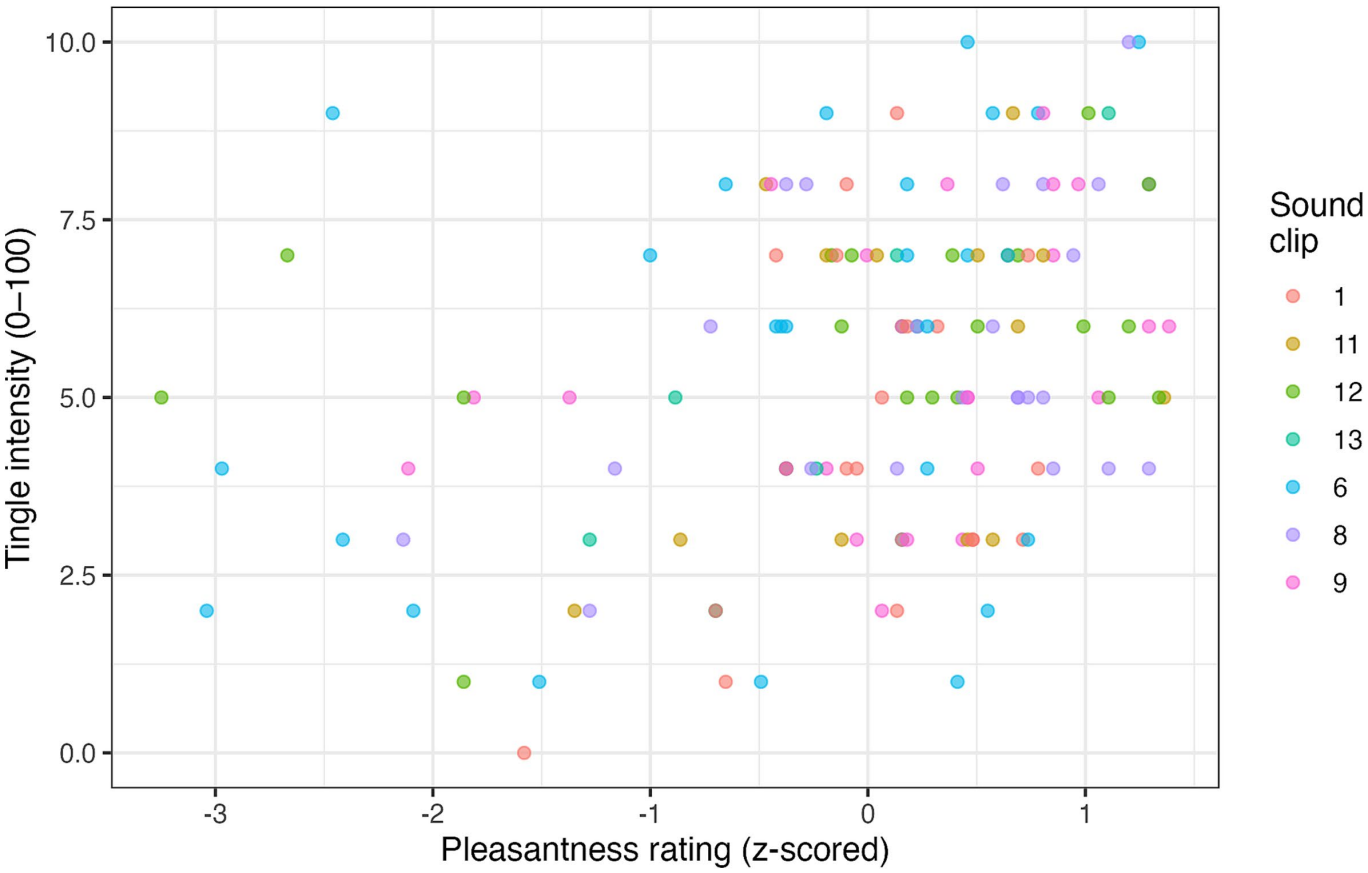
Scatter plot of the relationship between pleasantness (z-scored) and tingle intensity across 136 ASMR-positive trials. Each point represents one trial; colours indicate different sound clips.

On 136 ASMR-positive trials with self-rated intensity scores, tingle strength increased linearly with pleasantness (*β* = 1.06 ± 0.22, *t* = 4.71, *p* < 0.001), but not with arousal (*β* = −0.19 ± 0.16, *t* = −1.15, *p* = 0.25). Figure 3 shows the fixed-effect scatter plot, colour-coded by sound clip.

A follow-up interaction model revealed that pleasantness remained a strong positive predictor (*β* = 1.22 ± 0.23, *t* = 5.25, *p* < 0.001), while arousal was a significant negative predictor (*β* = −0.48 ± 0.20, *t* = −2.39, *p* = 0.018), and the Pleasantness × Arousal interaction also reached significance (*β* = 0.40 ± 0.18, *t* = 2.22, *p* = 0.028; see Table 2).

**Table 2:** Linear mixed-effects model of tingle intensity ratings (1–10 scale) on ASMR labelled trials.

Predictor	*β* (Estimate)	SE	df	*t*	*p*	95% CI (lower, upper)	Partial *R*^2^	95% CI (Partial *R*^2^)	Std. Beta (β*)
Intercept	4.51	0.31	24.8	14.33	<0.001	[3.86, 5.16]	–	–	–
Pleasantness (z)	1.22	0.23	126.0	5.25	<0.001	[0.76, 1.68]	0.145	[0.055, 0.262]	0.536
Arousal (z)	−0.48	0.20	119.3	−2.39	0.018	[−0.88, −0.08]	0.032	[0.001, 0.112]	−0.211
Pleasantness x Arousal	0.41	0.18	114.3	2.22	0.028	[0.04, 0.77]	0.027	[0.000, 0.103]	0.177

Predictors were z-scored pleasantness and arousal ratings from the affect grid, and their interaction. Random intercepts were included for participant and sound. Pleasantness was a strong positive predictor of tingle intensity, arousal showed a weak negative association, and the Pleasantness × Arousal interaction was significant. Fixed-effect estimates (*β*), SEs, dfs, *t*, *p*, 95% CIs, and partial *R*^2^ (95% CIs) are reported. Model fit: AIC = 589.59; BIC = 609.97; *R*^2^ (marginal) = 0.146; *R*^2^ (conditional) = 0.529; ICC = 0.448; RMSE = 1.40. Std. Beta (β*) from a refit with z-scored outcome.

This interaction suggests that the relationship between pleasantness and tingle strength became steeper when arousal was high. In other words, at higher arousal levels, pleasant sounds were more likely to elicit stronger tingles. Conversely, at low arousal, even pleasant sounds were less effective in producing high-intensity ASMR experiences. Interpreted on the original 0–10 scale, a 1 SD increase in pleasantness corresponded to an average +1.22 point increase in intensity. Effect sizes (partial *R*^2^) indicated unique contributions of pleasantness (partial *R*^2^ = 0.145, 95% CI [0.055, 0.262]), arousal (partial *R*^2^ = 0.032, 95% CI [0.001, 0.112]), and the interaction (partial *R*^2^ = 0.027, 95% CI [0.000, 0.103]). Model fit for the interaction model: AIC = 589.59; BIC = 609.97; *R*^2^ (marginal) = 0.146; *R*^2^ (conditional) = 0.529; ICC = 0.448; RMSE = 1.40. A fully standardised refit (z-scored intensity) yielded similar conclusions (Pleasantness β* = 0.536; Arousal β* = −0.211; Pleasantness × Arousal β* = 0.177, see Table 2).

### 4.3 Discussion in relation to the Proximity Prediction Hypothesis

These behavioural data align closely with key predictions of the PPH, which views ASMR as a vagal cascade triggered by a predicted social-touch event.

#### 4.3.1 Valence dominance

ASMR moments are defined by a large hedonic boost and only a minor arousal increase, this dissociation is exactly what would be expected if a slow-stroking CT-touch prediction drives the cascade while sympathetic output is actively suppressed, as the PPH predicts.

#### 4.3.2 ASMR experience

The logistic mixed-effects model revealed that pleasantness significantly predicted whether a trial was classified as ASMR, whereas arousal did not. The interaction between pleasantness and arousal was not significant, and model fit was not improved by its inclusion. These findings suggest that the ASMR classification decision relies primarily on the perceived reward value of the sound, a direct prediction of the PPH, and is largely unaffected by concurrent arousal levels.

#### 4.3.3 Intensity gradient

Within trials when ASMR was reportedly experienced in response to the sound, the intensity of tingles increased with pleasantness. Furthermore, an interaction emerged where pleasantness was an even stronger predictor of tingle strength when arousal was high. Under the PPH, this fits the notion that tingle intensity is a graded posterior-insula simulation of predicted touch value, with arousal acting as a gain control mechanism. That is, when arousal is elevated, the system may amplify the hedonic signal, but only when that signal is already strong. These data align with recent behavioural evidence from Gillmeister et al. (2022), who found that tingle intensity during ASMR closely tracked pleasantness and was further amplified by interpersonal touch. This could suggest that affective valence is central to the ASMR simulation, while arousal may act as a gain control mechanism, steepening the link between high pleasantness cues and tingling under certain conditions.

#### 4.3.4 Conclusion

Taken together, the data suggest that hedonic valence is the primary driver of both ASMR occurrence and intensity. Arousal shows a more equivocal role, sometimes enhancing the pleasantness-tingle gradient, but otherwise exerting weak or inconsistent effects. This ambiguity fits with the PPH view that arousal may reflect both proximity-based alerting and vagally mediated suppression, depending on the listener and context. Nevertheless, the data must be interpreted cautiously. First, the retrospective survey design may not capture tingles with the precision of real-time reports. Second, the undergraduate sample limits generalisability, and the exclusion of mouth sounds reduces ecological validity. Finally, while EEG data were collected in the broader study, no neural analyses are reported here; instead, this behavioural dataset is intended to provide illustrative, hypothesis-testing support for the PPH, with complementary EEG and MEG findings by the authors to be addressed in an upcoming study. The remaining discussion in this paper will cover the clinical applications and other proposed future tests of the PPH.

## 5 Current EEG evidence in relation to the PPH

While the present paper focuses on illustrative behavioural data, as the PPH is a predictive coding account that posits specific cortical dynamics, it is also important to situate it within the context of existing EEG findings on ASMR. Although the literature is still limited and heterogeneous in methods, several converging results speak to the neural plausibility of the PPH cascade, and can be assessed with regards to conventional frequency bands: alpha ≈ 8–12 Hz; sensorimotor rhythm, SMR ≈ 12–15 Hz; beta ≈ 15–30 Hz; gamma ≥ 30 Hz (Schomer and Lopes da Silva, 2018). The PPH anticipates that when a near-ear cue engages peripersonal space and the system begins to simulate CT-optimal touch in posterior insula and secondary somatosensory cortex, beta activity over somatosensory/posterior insular regions would decrease as an index of active sensory processing. If the prediction is then accepted and integrated as a result of the individual’s priors, a rise in gamma band power may reflect precision-weighted updating of the interoceptive state. As the system settles into parasympathetic calm, alpha and SMR could increase, consistent with sensorimotor quieting and relaxed alertness. Transient alpha reductions at the outset would also be compatible with early sensory analysis, so an assessment of the response’s temporal profile is critical for future research.

Viewed through this lens, the heterogeneous EEG literature becomes more interpretable without being committed to a single outcome. Several reports align with the hypothesised updating-and-settling biphasic PPH cascade. Fredborg et al. (2021) found increases in alpha, gamma, and SMR in ASMR experiencers relative to non-experiencers during auditory triggers, patterns that could reflect precision updating (gamma) followed by sensorimotor quieting (alpha/SMR). Lee et al. (2020) similarly observed increases in SMR, alpha, and gamma for ASMR compared with binaural beats, suggesting a shift beyond simple drowsiness. Ohta and Inagaki (2021) reported that when cognitive load suppressed alpha and elevated high-beta/gamma, exposure to ASMR stimuli moved alpha and gamma/high-beta back toward resting levels, which may indicate re-balancing and parasympathetic calm once the prediction is accepted.

Other findings appear more consistent with earlier stages of processing. Engelbregt et al. (2022) reported reductions in alpha and theta with elevated beta, including alpha decreases over temporal–parietal sites, which could reflect early sensory analysis and the initial orienting phase in posterior temporal–parietal regions involved in audio-tactile integration. Seifzadeh et al. (2021) likewise observed alpha reductions during ASMR video viewing, again consistent with an initial engagement phase when first alerting to the stimuli, rather than the later parasympathetic settling phase. Regionally, Koo et al. (2021) showed that ASMR and control videos diverge in gamma modulation over occipital and central sites, compatible with cross-modal recruitment of sensory networks. Pedrini et al. (2021) identified distinct spectral signatures across baseline, relaxed, and ASMR states, implying large-scale network shifts that might be expected when the system transitions from orienting into affiliative calm.

Taken together, current EEG findings do not yet provide a single, time-resolved demonstration of the full sequence of events involved in the proposed PPH cascade. Nevertheless, recurrent reports of gamma and SMR modulation, alpha changes, and posterior temporal–parietal involvement are compatible with key stages suggested by the PPH model. A decisive test now calls for time-locking analyses to reported tingle onsets, source-localised EEG or MEG focusing on posterior insula/OP1-S2 and posterior STS regions, and concurrent autonomic indices such as measures of high-frequency HRV and pupil diameter. If the PPH is a plausible explanation for the mechanism behind the ASMR phenomenon, future work should observe a stepwise pattern in which early beta reductions in somatosensory/post-insular regions are followed by a gamma increase in the posterior insula and, subsequently, an alpha/SMR up-shift consistent with sensorimotor quieting, with the magnitude of these changes covarying with vagal markers. Critically, this sequence is falsifiable: a failure to observe the predicted timing, regional specificity, or coupling with autonomic measures would argue against the PPH account. Suggestions for this kind of future research are discussed in Section 7.4 of this paper.

## 6 Integrating PPH with CNS-ANS communication and clinical angles

The Proximity Prediction Hypothesis (PPH) describes how a near-ear sound can initiate a cascade that ends in tingles and calm. The present section places that mechanism within wider brain–body communication processes, details the existing physiological clues that suggest the proposed PPH chain is real, and explains why the same mechanism could become a cheaper, accessible alternative to treatments like vagus nerve stimulation (VNS), particularly valuable for anxious and autistic populations as well as though suffering from sleep issues. VNS refers to the implanted, pulse generator therapy in which electrodes are wrapped around the cervical vagus to deliver periodic electrical bursts, a treatment approved for drug-resistant epilepsy and difficult to treat depression already.

### 6.1 Parallels with transcutaneous auricular vagus nerve stimulation and its clinical benefits

Electrical transcutaneous auricular vagus nerve stimulation (taVNS) is a wearable version of implanted VNS. Instead of placing electrodes on the cervical vagus, two small clip electrodes are positioned on the cymba conchae, this is the upper hollow of the outer ear where the auricular branch of the vagus nerve (ABVN) terminates in the skin. A battery-powered stimulator then delivers painless, low frequency pulses (typically 25 Hz, 200–300 μs) for about 15 min. Because the ABVN projects directly to the nucleus tractus solitarius (NTS) in the brainstem, the current accesses central vagal pathways without passing through major muscle or bone tissue, unlike cervical VNS. From the NTS the signal ascends to the LC and parabrachial complex and descends to the dorsal motor nucleus of the vagus (DMV), shifting the LC to DMV balance toward parasympathetic dominance. The immediate physiological signature (heart rate deceleration and a rise in high frequency HRV) has been reported to appear within five minutes of stimulation (Borges et al., 2021) and mirrors the pattern ASMR listeners report during tingles.

The current model proposes that taVNS offers a useful clinical precedent for what the PPH suggests ASMR may achieve through sensory prediction. However, important constraints must be acknowledged. Unlike taVNS, ASMR cannot guarantee stimulation of the auricular branch of the vagus, and responsiveness to ASMR varies considerably across individuals. Thus, while the analogy might be compelling, its translational potential should be understood as conditional on ASMR susceptibility.

Controlled trials have demonstrated that nightly sessions of taVNS significantly enhance sleep quality, reduce insomnia severity, and increase total sleep duration in individuals with chronic insomnia (Zhang et al., 2024; Wu et al., 2022). Similarly, heart rate deceleration of 3–5 bpm and a 5–8% HF-HRV gain has been found during reported ASMR tingling episodes (Poerio et al., 2018) and around 80% of habitual listeners use ASMR to fall asleep (Barratt and Davis, 2015).

Recent studies have demonstrated that brief taVNS courses translate the vagal tone shift into clinically meaningful anxiety relief. In a double-blind, randomised controlled trial, Ferreira et al. (2024) found that a brief taVNS protocol significantly reduced anxiety symptoms in university students, as measured by the Beck Anxiety Inventory, with effects persisting up to 2 weeks after stimulation. A recent randomised clinical trial by Zhang et al. (2024) found that 8 weeks of taVNS significantly reduced anxiety and depression symptoms, as measured by the Hamilton Anxiety Scale (HAMA) and Hamilton Depression Scale (HAMD) alongside significantly improved Pittsburgh Sleep Quality Index (PSQI) scores. These studies confirm that taVNS can pivot the LC-DMV axis from sympathetic vigilance toward parasympathetic calm and that standard clinical measures of sleep, anxiety, and depression offer realistic indices of this shift. Eid et al. (2022) found that ASMR-experiencers, who began with higher baseline state anxiety, experienced a significant reduction in State–Trait Anxiety Inventory-State subscale (STAI-S) scores after viewing an ASMR video, while non-experiencers did not.

Taken together, both taVNS and ASMR appear to converge on a common LC-DMV pathway, but by different routes: taVNS through exogenous current, and ASMR through a sensory prior that “pleasant CT-touch is imminent”. The PPH therefore predicts that the magnitude of an individual’s ASMR-induced HF-HRV burst should correlate with sleep and anxiety improvements, but such effects will depend on whether the person is an ASMR responder.

Future work can evaluate this prediction with single night polysomnography and standard anxiety inventories such as STAI-S. Currently, taVNS is being trialled as an intervention for treatment-resistant depression, PTSD and insomnia, yet it requires specialised hardware and clinical monitoring. ASMR could offer a headphone based, low cost, surrogate for taVNS, potentially expanding vagal tone interventions to populations who lack access to medical hardware, if listeners do experience meaningful levels of ASMR from the chosen stimuli, potentially providing similar clinical benefits that anyone with headphones could utilise.

### 6.2 Why anxious and autistic listeners might benefit most from ASMR-based interventions

A growing evidence base confirms that listeners do not seek out ASMR videos merely for curiosity or entertainment but because the experience delivers measurable relief from anxiety and sleeplessness, ASMR is therefore ripe with potential clinical applications.

How can the predictive coding mechanism underpinning PPH further hone ASMR’s clinical applications to specific populations? Both anxiety disorders and autism spectrum conditions are thought to be characterised by fundamentally over-precise interoceptive prediction errors (Paulus and Stein, 2006; Pellicano and Burr, 2012). In functional terms the insula “cries wolf,” keeping LC tone elevated and vagal tone low. A parallel Bayesian account proposes that autistic brains under-weight priors and over-weight sensory evidence, forcing even mundane events to register as surprising and arousal-worthy (Pellicano and Burr, 2012; Lawson et al., 2014). Both scenarios keep the insula-LC loop chronically engaged. From this perspective, PPH generates the hypothesis that individuals with higher baseline LC tone (such as those with anxiety or autistic traits) may show a larger dynamic range for LC suppression during ASMR, and thus greater HF-HRV gains and stronger subjective relief. This remains to be tested; future studies could compare autonomic responses and symptom reductions in anxious, autistic, and neurotypical groups during ASMR exposure, using metrics such as HF-HRV, pupil dynamics, and validated anxiety scales.

Reported experiencers of ASMR have been shown to score higher on neuroticism and anxiety than non-experiencers, suggesting they may have more to gain from a parasympathetic tilt in general (McErlean and Banissy, 2017). Supporting this, Poerio et al. (2022) demonstrated that ASMR experiencers exhibit heightened sensory sensitivity across multiple modalities, including increased bodily awareness and interoceptive sensitivity. Autistic individuals often display atypical interoception; atypical emotional clarity, alexithymia, and interoceptive confusion (Bonete et al., 2023). Sensory processing in autism is also frequently atypical, with both hyper- and hypo-responsiveness across modalities (Elwin et al., 2013), possibly accompanied by somatosensory amplification. Interestingly, some autistic adults report heightened bodily awareness despite reduced interoceptive accuracy, indicating a mismatch between subjective and objective interoceptive states (Garfinkel et al., 2016). This convergence suggests that ASMR may be especially impactful for individuals with enhanced sensory and emotional responsiveness, although responses will likely vary depending on how the social and affiliative meaning of ASMR stimuli is interpreted. For some autistic individuals, the social cues embedded in whispers or gaze may not carry the same affiliative value, which could reduce ASMR efficacy. Future studies should therefore stratify participants by both sensory sensitivity and social priors.

While ASMR proneness also correlates with trait empathic concern (McErlean and Banissy, 2017), this does not preclude its relevance for autistic individuals, who may differ in “cognitive empathy”, i.e., imagining another person’s mental state, but not necessarily “affective empathy”, the capacity to emotionally resonate with affiliative or caring cues (Dziobek et al., 2008). These findings map onto the PPH cascade: a powerful, but non-intrusive, prior, silences insular error signals, drops LC tone, and brings the body into a parasympathetic state that many autistic and anxious individuals may otherwise struggle to access. If near-ear audio can normalise the LC-DMV balance in these populations, it may again serve as a low-cost alternative to taVNS, especially for children or adults who are needle-averse or have restricted access to neurostimulation clinics, but its clinical utility will depend on individual responsiveness and the interpretation of the sensory cues.

Evidence from tactile research reinforces the clinical logic. Even a single session of massage, can produce immediate reductions in state anxiety, along with decreases in blood pressure and heart rate (Moyer et al., 2004). Scalp massage specifically, in office workers, significantly reduced cortisol, blood pressure, heart rate, and self-reported stress (Kim et al., 2016), suggesting that even brief, localised tactile input can rapidly shift the autonomic balance toward parasympathetic dominance. Yet CT-touch is not always socially available or desired by people with heightened sensory sensitivities. ASMR supplies a predictive, contact-free analogue that can be self-administered with nothing more than headphones for people in anxious or autistic populations who may be otherwise touch-avoidant.

Crucially, autistic individuals often show altered tactile sensitivity and hedonic perception to stimuli targeting CT-innervated regions (Cascio et al., 2008), with neuroimaging further indicating that CT-evoked responses in social brain regions such as the orbitofrontal cortex and superior temporal sulcus are diminished in individuals with higher autistic traits (Voos et al., 2013), while EEG work shows that neural potentials to CT-targeted touch scale negatively with autistic trait load (Haggarty et al., 2020). Moreover, the coupling between subjective reports of pleasantness and central neural representations of touch has previously been found to be weaker in samples of adolescents with autism, suggesting a disconnect between afferent input and hedonic experience (Perini et al., 2021). Taken together, these findings imply that while CT afferents may be intact, their central processing and translation into pleasant affect is atypical in autism. This supports the novel possibility that auditory ASMR cues, which deliver the prediction of affiliative contact without relying on CT-fibre stimulation, could bypass these atypical responses and more effectively evoke pleasantness and parasympathetic calming. Although this remains to be tested directly, it highlights a potential route by which ASMR might provide sensory-affective benefits to autistic individuals even where CT-touch itself is less effective.

Notably, not all individuals with anxiety or autism may benefit equally from ASMR cues that mimic CT-optimal light stroking. For those with atypical CT processing, auditory ASMR may bypass tactile deficits and still evoke affiliative priors, as argued above. However, other evidence suggests that some anxious or autistic individuals instead find deep pressure touch more calming than light touch, with studies of weighted blankets and squeeze devices showing reductions in arousal, anxiety, and insomnia in certain responders (Grandin, 1992; Edelson et al., 1999; Ekholm et al., 2020; Fava et al., 2021). Within the PPH framework, this raises a distinct, testable hypothesis: for individuals less responsive to CT-mimetic ASMR, auditory cues that mimic the sensory qualities of deep pressure, such as low-frequency, steady, broadband sounds, may better initiate a vagal release and lead to parasympathetic calm. This refinement does not imply that all ASMR works via multiple routes, but rather that individual differences in tactile preference may determine which acoustic simulations are likely to be effective given individual differences. Future work can therefore stratify participants by CT sensitivity and deep-pressure preference to identify which subgroups might benefit most from which classes of ASMR stimuli.

#### 6.2.1 Exploratory clinical protocol

A logical next step is to evaluate ASMR in structured clinical trials using designs comparable to those employed in taVNS research. A preliminary protocol could involve nightly exposure to a curated ASMR playlist, delivered via headphones, for 15–20 min before sleep over a period of 4–8 weeks. These parameters deliberately mirror the taVNS insomnia trials mentioned earlier in this report, which used sessions of up to 30 min, within multi-week courses; preserving the pre-sleep timing and cumulative dosing window (Wu et al., 2022; Zhang et al., 2024). Participants would complete validated measures of anxiety (e.g., STAI-S), depression (HAMD), and sleep quality (PSQI), alongside autonomic monitoring (HF-HRV, pupillometry) in a subset of sessions. Long-term follow-up (e.g., 1–3 months later) could test the durability of effects using the same clinically meaningful measures, while stratifying participants by ASMR susceptibility would identify which subgroups (e.g., anxious, autistic, or neurotypical) derive the greatest benefit. Such a design would provide a concrete test of ASMR’s translational potential, clarifying both its efficacy and its boundary conditions.

### 6.3 Refining new CAN biomarkers

The PPH model lends itself to proposing two straightforward improvements to the resting HF-HRV score as a biomarker of CAN health, which dominates the current literature. Firstly, instead of looking at HRV in a long, resting baseline, how it changes from trial to trial could be observed while someone is listening to ASMR. A mixed-effects regression of HF-HRV gain and reported tingle intensity will provide a slope for each person. A steep positive slope should indicate that the person’s vagus nerve immediately answers the brain’s “this is pleasant and safe” signal; a flat slope means it does not. If the PPH model is correct, the individual differences in responsiveness are possibly more informative about anxiety risk or sleep quality for that individual than a single resting HF-HRV snapshot. Furthermore, if the PPH is supported, then combining biomarkers like beta band power decreases in the posterior insula (suggesting the system in PPH is predicting a gentle CT-touch is about to happen), along with HF-HRV gain, would provide a mechanistically coherent biomarker that directly indexes the hypothesised cascade from cortical prediction to autonomic change. Such a multimodal index could predict who will report feeling less anxious or who might fall asleep faster, more reliably than either brain or heart signal could when taken on its own.

In short, the PPH model suggests that ASMR tingling could be used as a convenient stress-test of the CAN loop across individuals, one that can be quantified in real time and may add to the current diagnostic toolkit for anxiety, insomnia, and related conditions. Further experimental paradigms that could be used to test the PPH model are discussed in the next section.

## 7 Future tests of the PPH model

The PPH makes concrete, falsifiable claims about where in the sensory chain the ASMR cascade begins and how it propagates through the insula-LC-vagus axis. Below, a series of experimental predictions, ranging from psychophysics to source-localised MEG, are outlined, to suggest what results would support these claims in future research.

### 7.1 Distance manipulation predictions

#### 7.1.1 Binaural morphing of approach cues

The PPH model suggests that the ASMR cascade is gated by perceived proximity, such that a continuous morphing of binaural cues from far (>1 m) to near field (<30 cm) should show a non-linear inflection point in ASMR reports, with tingle likelihood, pleasantness, and vagal markers (e.g., HF-HRV gain) rising sharply as the sound enters the peri-aural space. This would reflect the transition into the brain’s peripersonal comfort zone, aligning with prior PPS boundaries observed in audio-tactile studies (Ferri et al., 2015; Serino et al., 2009). Given that pupil dilation has been observed during ASMR listening, likely reflecting heightened attentional engagement with the sound, the PPH further predicts that a delayed pupil constriction should follow as parasympathetic dominance increases during the latter stages of the response. This later-phase constriction has not yet been empirically tested but would be expected if the LC-vagus balance shifts toward sustained calm. This could be tested using interaural time/level difference manipulations of a typically ASMR-inducing stimulus, such as “realistic haircut sounds” (Schürmann et al., 2006).

#### 7.1.2 Disrupting spatial coherence across ears

If spatial proximity is integrated across both ears to determine whether the stimulus is near or far, then presenting conflicting distance cues across ears (e.g., one ear hears a close whisper; the other a far-filtered version) should reduce ASMR responses and vagal activity, relative to conditions with coherent near-field input in both ears. This would support the view that the brain uses spatial coherence as a gating signal for engaging the insula-LC-vagus cascade and disrupting it should reduce the probability of experiencing tingles.

### 7.2 Combining real CT-touch with near-ear audio predictions

Recent findings by Gillmeister et al. (2022) indicate that ASMR responders not only exhibit a higher incidence of mirror-touch synaesthesia but also report greater positive emotional reactions to social touch, especially those with stronger ASMR traits. While this supports the notion that affective touch and ASMR share common hedonic mechanisms, the next step is to test whether these effects reflect underlying prediction-based neural dynamics. If the PPH is correct, combining real CT-touch with auditory cues should produce distinct physiological and neurophysiological signatures that reflect audio-tactile congruence and temporal precision.

#### 7.2.1 Audio-tactile congruence

Stroking of the listener’s scalp at CT-optimal velocity (3 cm s^−1^) while presenting either a near-ear brushing sound (congruent) or an identical sound filtered to far-space (incongruent) should boost posterior-insula *β*-ERD and HF-HRV if the PPH is to be supported. Whereas incongruence will dilute both markers, because the prediction error becomes more precise when the auditory prior and tactile evidence disagree (Ellingsen et al., 2016).

### 7.3 Expectation modulation and proximity cue predictions

Ellingsen et al. (2013) devised an elegant “placebo-hedonia” protocol; an inert nasal spray presented as a “pleasure enhancer”, followed by slow brush strokes on the forearm during fMRI. The placebo increased subjective pleasantness ratings by around 25%, with enhanced BOLD activity in S1, S2, and the posterior insula, and elevated functional coupling between the pregenual ACC (pgACC) and periaqueductal gray areas, supporting a top-down prediction-based modulation of somatosensory gain. In predictive coding terms, the positive label increased the precision of the “this will feel good” prior, allowing top-down signals to dominate and turn up the gain on the incoming CT volley.

Building on Ellingsen’s finding that positive expectancy amplifies CT-touch processing, if the PPH is correct in asserting that tingle cascades result from precision-weighted predictions of CT-optimal touch, then positively framing a binaural track (e.g., labelling it as a “clinically validated tingle inducer”) should increase posterior-insula *β*-band desynchronization, enhance vagal tone (HF-HRV), lead to a constriction in tonic pupil diameter, and raise subjective ratings of tingle intensity and pleasantness, provided the track contains proximal, near-earl spatial cues. Furthermore, if spatial proximity is a prerequisite for CT-touch predictions, then far-filtered versions of the same track should fail to elicit ASMR responses, even under positive expectancy conditions. That is, labelling alone will not boost tingles or parasympathetic markers when the sensory input lacks coherent proximity information. This prediction sharply distinguishes the PPH from a purely cognitive account: both sensory proximity and cognitive framing must converge to silence prediction error and initiate ASMR.

### 7.4 Predicted EEG and MEG signatures of the PPH cascade

If the PPH is correct, ASMR should elicit a specific neural-autonomic sequence reflecting affective touch simulation and vagal modulation. Empirically, EEG studies show that ASMR triggers produce increased alpha, gamma, and modulations in sensorimotor rhythms (Fredborg et al., 2021) and reduced theta coupled with elevated beta (Engelbregt et al., 2022), along with immediate pupil dilation (that the PPH model would suggest relates to the proposed initial orienting stage) during strong ASMR episodes (Pedrini et al., 2021). Building on this, PPH predicts a time-locked cascade in the EEG: an initial beta-band suppression over centroparietal sites reflecting S2/posterior-insula activation for CT touch, followed by a transient gamma enhancement indexing precision-weighted updating. Later increases in beta reported in some studies may correspond to regulatory or arousal-related processes rather than the initial sensory stage. Time-resolved EEG and source-localised MEG are therefore crucial to test whether early beta decreases and later gamma increases can be distinguished in real ASMR episodes. MEG, with better spatial resolution, should localise this beta-gamma sequence to the posterior insula and OP1/S2, with earlier beta suppression in pSTS marking peripersonal space detection, and elevated beta-band coherence between the posterior insula and pgACC/vmPFC regions during the tingling window (reflecting precision-weighted prediction). Crucially, stronger posterior-insula beta suppression should correlate with larger increases in high frequency heart rate variability (HF-HRV), supporting the proposed insula-LC-DMV coupling underlying vagal gain in ASMR. These neural-autonomic patterns should be absent or markedly reduced in control trials without reported tingles, or when identical stimuli are presented with far-field spatial filtering, providing a decisive test of PPH.

If this mechanism is supported in future work, ASMR videos could evolve from quirky bedtime rituals into evidence-based, widely accessible therapeutic interventions for anxiety reduction and sleep promotion. This is especially salient for populations such as those with autism, where prediction error is chronically elevated and conventional relaxation techniques often fail. Moreover, the proposed neural-autonomic markers of beta suppression in the posterior insula, HF-HRV gain, and pupil constriction, could serve as future biomarkers for personalised treatment selection and efficacy tracking. Each paradigm offered in the future research section isolates a different link in the proposed chain; proximity detection, CT-touch prediction, LC suppression, and vagal release. Convergent success across distance manipulation, expectancy modulation, and longitudinal outcome trials would transform PPH from a heuristic into a mechanistically validated account of ASMR and, by extension, into a blueprint for audio-based vagal therapies in mental health. By explicitly integrating predictive coding principles with the neurophysiology of interoception, PPH also offers a broader contribution to our understanding of how the brain regulates the body in response to socially salient sensory cues.

## 8 Conclusion

The Proximity Prediction Hypothesis does more than explain an unusual, pleasant tingling sensation; it places the ASMR phenomenon within the LC-vagus system that modern affective neuroscience regards as influential to various physiological and neurological functions, including emotional regulation, stress responses, and even cognitive abilities. Near-ear sounds appear capable of fooling the brain, leading to emotional modulation, where a sensory cue suppresses the noradrenergic accelerator (the LC), allowing disinhibition of the vagal brake, and ushers both the brain and body into a restful state. The PPH therefore provides a predictive-coding framework specifying when and how the plausible neural routes proposed by previous structural accounts, such as McGeoch and Rouw’s (2020) cross-activation model, might be engaged, and how the characteristic tingling experience could be generated as a result.

The illustrative data reported here offer behavioural support for this framework. Across trials, ASMR experiences were strongly predicted by hedonic valence (pleasantness), not by physiological arousal, and tingle intensity scaled with pleasantness, in a manner that was modestly amplified by arousal. These patterns are consistent with the PPH account of ASMR as a reward-based simulation of safe affective proximity, rather than a state of heightened energetic activation.

In summary, the Proximity Prediction Hypothesis situates ASMR within predictive coding accounts of interoception, offering a mechanistic framework that links acoustic cues, tingling sensations, and parasympathetic calming. The behavioural data reported here are consistent with this account, though they remain preliminary. Rather than providing definitive empirical validation, the present study illustrates how PPH can integrate existing autonomic and behavioural findings into a coherent model, and points toward future work needed to directly test its neural predictions.

## Data Availability

The raw data supporting the conclusions of this article will be made available by the authors, without undue reservation.
